# Supplementary material for: Participatory research towards the control of snakebite envenoming and other illnesses in a riverine community of the Western Brazilian Amazon
Source: PLoS Negl Trop Dis. 2025 Jan 23;19(1):e0012840. doi: 10.1371/journal.pntd.0012840 (PMC11793770; doi:10.1371/journal.pntd.0012840)
Supplement: S1 File — (PDF) [file pntd.0012840.s001.pdf]

S1. File. Socio-demographic questionnaire

Sociodemographic Data ***Date of Collection: ____/______/______***

Name: ____ Gender: ( ) Female ( ) Male

Date of Birth:____/____/_____ Place of Birth::_______________________

Marital Status: ( ) Single ( ) Married ( ) Divorced ( ) Widowed

Education Level: ( ) 1 to 5 years ( ) 6 to 10 years ( ) 11 to 15 years ( ) More than 15 years

Current Occupation: ( ) Homemaker ( ) Farmer ( ) Fisherperson ( ) Unemployed ( ) Teacher ( ) Other: ______________

1. **What is your religion?**
   A) Catholic
   B) Evangelical
   C) Jehovah's Witness
   D) Spiritist
   E) No religion
   F) Other: _________

2. **Type of housing:**
   A) Wood
   B) Brick
   C) Floating
   D) Stilt house
   E) Other: ___________

3. **What items are in the house? ***
   A) Electricity
   B) Radio
   C) Television
   D) Landline
   E) Stove
   F) Iron
   G) Kerosene lamp
   H) Refrigerator
   I) Freezer

4. **What is the main source of drinking water?**
   A) Public pipeline
   B) Private well
   C) Community well
   D) Surface water/stream
   E) Water tank supply
   F) Rainwater
   G) Other: ________

5. **Type of sanitary facilities:**
   A) Community latrine/toilet outside the house
   B) Private latrine/toilet outside the house
   C) Riverbank
   D) Private bathroom inside the house
   E) Other: ________

6. **Do you own any of the following items? ***
   A) Motorcycle
   B) Car
   C) Bicycle
   D) Rowboat
   E) Motorized canoe
   F) Other: ____________

7. **Household income source: ***
   A) Social Security (INSS)

8. **Total household income:**
   A) Up to R$ 500.00
   B) Up to R$ 1,100.00
   C) Up to R$ 2,200.00
   D) Up to R$ 3,300.00
   E) More than R$ 3,300.00

9. **How many people live in the house?**
   A) Up to 3 people
   B) Up to 5 people
   C) Up to 7 people
   D) Up to 10 people
   E) More than 10 people

10. **Do you own a cellphone?**
    A) Yes
    B) No

11. **Do you have access to a computer or tablet?**
    A) Yes
    B) No

12. **Do you have internet access?**
    A) Yes
    B) No

13. **If yes, how many hours per day?**
    A) Less than 1 hour per day
    B) Between 2-3 hours per day
    C) Between 4-5 hours per day
    D) Between 6-7 hours per day
    E) More than 7 hours per day

14. **Social networks:**
    A) Facebook
    B) Instagram
    C) WhatsApp
    D) Twitter
    E) Other: ________

15. **What is the most serious disease in the region?**
    A) Dengue
    B) Malaria
    C) Hepatitis
    D) Parasitic infections
    E) Snakebite
    F) Viruses causing diarrhea and vomiting
    G) Other: ________

B) Family Grant (Bolsa Família)<br>C) Formal employment (CLT)<br>D) Self-employment<br>E) Public servant<br>F) Daily labor<br>G) Other: ________		
Behaviors and risks	***Yes***	***No***
Do you engage in fishing, farming, or hunting activities?<br>If yes, how often?<br>A) Up to 2 times per week<br>B) Up to 5 times per week<br>C) Up to 7 times per week		
Have you ever put your hand into holes, termite mounds, or tree trunks?		
Do you wear closed high boots?		
Do you use thick gloves to handle dry leaves, piles of garbage, firewood, and palm leaves?		
Do you usually pay attention to the path you are walking?<br>A) Always<br>B) Sometimes<br>C) Never		
Do you jump directly over logs, branches, and leaves?		
Do you usually walk barefoot?		
* If yes, where?<br>A) Indoors<br>B) In the backyard<br>C) On the street<br>D) Other: ________		
Do you usually seek medical assistance when you are sick?		
* If yes, where?<br>A) Basic Health Unit (UBS)<br>B) Emergency Care Unit (UPA)<br>C) Hospital<br>D) Other: _________		
In the early morning or late afternoon, do you perform any activities near vegetation?<br><br>If yes, which ones? ______________________________		
Have you ever encountered a snake?		
If yes, how many times?<br>A) 1<br>B) 2<br>C) 3<br>D) 4<br>E) 5 or more		
Is there a bushmaster snake (Lachesis muta) in the community?		
Is there a rattlesnake in the community?		
Is there a coral snake in the community?		
Is there a jararaca snake (Bothrops) in the community?		
Do you know how to identify venomous snakes?		
Is a snakebite a serious health problem?		
Are snakebites a health problem that requires hospital treatment?		
Does a tourniquet help treat a snakebite?		
Has any family member ever been bitten or stung by an animal?		
If yes, which one?<br>A) Stingray<br>B) Spider<br>C) Scorpion<br>D) Snake<br>E) Tucandeira ants (bullet ants)		

F) Caterpillars<br>G) Others: __________<br><br>If yes, who?<br>A) Mother<br>B) Father<br>C) Sister/Brother<br>D) Spouse<br>E) Son/Daughter<br>F) Other: __________		
Which body part was affected?<br>A) Head<br>B) Torso<br>C) Arm<br>D) Forearm<br>E) Thigh<br>F) Leg<br>G) Feet<br>H) Hands<br><br>What was the outcome?<br>A) Recovery<br>B) Death<br>C) Sequelae (long-term effects)	What was the local manifestation?<br>A) Edema (swelling)<br>B) Redness<br>C) Necrosis (darkened tissue)<br>D) Inflammation (pus)<br>E) Vomiting<br>F) Blood in the nose/mouth<br>G) Blood in the urine<br>H) Chills<br>I) Seizure<br>J) Fever<br>K) Other: ________	
Have you ever been bitten or stung by a venomous animal?		
If yes, which animal?<br>A) Stingray<br>B) Spider<br>C) Scorpion<br>D) Snake<br>E) Tucandeira ants (bullet ants)<br>F) Caterpillars<br>G) Other: ________<br><br>If yes, how many times?<br>A) 1<br>B) 2<br>C) 3<br>D) 4<br>E) 5 or more<br><br>If yes, which body part was affected?<br>A) Head<br>B) Torso<br>C) Arm<br>D) Forearm<br>E) Thigh<br>F) Leg<br>G) Feet<br>H) Hand	What were the local manifestations?<br>A) Edema (swelling)<br>B) Redness<br>C) Necrosis (darkened tissue)<br>D) Inflammation (pus)<br>E) Vomiting<br>F) Blood from the nose/mouth<br>G) Blood in the urine<br>H) Chills<br>I) Seizures<br>J) Fever<br>K) Other: ________<br><br>What was the outcome?<br>A) Recovery<br>B) Death<br>A) C) Sequelae (long-term effects)	
What was used in the treatment?<br>A) Antibiotic<br>B) Pain reliever/anti-inflammatory/antipyretic<br>C) Specific prescription<br>D) Herbal decoction from tree bark or leaves<br>E) Alligator or snake fat<br>F) Antivenom/antidote<br>G) Prayer<br>H) Amulet<br>I) Other: ________		
Did you receive medical care?		
If yes, where?<br>A) Basic Health Unit (UBS)<br>B) Emergency Care Unit (UPA)<br>C) Hospital	If not, what was the reason?<br>A) Did not think medical care was necessary<br>B) Could not reach the healthcare facility in time<br>C) Refused to be taken to a healthcare service	

D) Other: ________<br>How was the care provided?<br>A) Terrible<br>B) Poor<br>C) Good<br>D) Excellent	D) Lacked financial resources or transportation<br>E) Prioritized treatment with traditional medicine<br>F) Other: _______________	
Do you know if antivenom was administered for the bite?		
Did you have any sequelae after the incident?		
If yes, which one?<br>A) Amputation<br>B) Extensive scarring<br>C) Reduced movement<br>D) Loss of movement<br>E) Disability caused by the incident<br>F) Other: ________		
