# Supplementary material for: Participatory research towards the control of snakebite envenoming and other illnesses in a riverine community of the Western Brazilian Amazon
Source: PLoS Negl Trop Dis. 2025 Jan 23;19(1):e0012840. doi: 10.1371/journal.pntd.0012840 (PMC11793770; doi:10.1371/journal.pntd.0012840)
Supplement: S2 File — (PDF) [file pntd.0012840.s002.pdf]

S2. File. Previous knowledge and experience about SBE

Name: ***Data of collection:*** ____/______/______

Prevention/Treatment/First Aid

1. **What objects PROTECT against snakebite?**
   A) Flip-flops
   B) Long pants
   C) Jeans
   D) Rubber gloves
   E) Boots
   F) Flashlight
   G) Amulet
   H) I don't know
   I) Others: ___________

2. **What should be done for a snakebite?**
   A) Apply a tourniquet
   B) Cut the bite area and suck the venom
   C) Apply urine
   D) Apply herbs
   E) Go to the hospital
   F) Seek the leader
   G) Pray
   H) I don't know
   I) Others: ___________

3. **If an accident happens to you or a family member now, what would you do?**
   A) Wait for symptoms to appear
   B) Use medicines I have at home
   C) Go to the hospital immediately
   D) Seek the leader immediately
   E) Go to the UBS (Basic Health Unit) immediately
   F) Other action: ______________

4. **What is used for a snakebite? ***
   A) Antibiotic
   B) Pain reliever/anti-inflammatory/antipyretic
   C) Specific prescription
   D) Herbal decoction from tree bark or leaves
   E) Alligator fat
   F) Snake fat
   G) Homemade serum
   H) Antivenom/antidote
   I) Tea
   J) I don't know
   K) Others: ___________

5. **If an accident happens to you or a family member now, would you wait for symptoms to appear before seeking help?**
   A) Yes
   B) No
   C) I don't know

6. **What symptoms do you think will appear after a snakebite? ***
   A) Pain
   B) Edema (swelling)

C) Blood from the nose/mouth
D) Blood in the urine
E) Chills
F) Convulsions
G) Fever
H) Vomiting
I) Others: _______________

7. Can the person who was bitten eat "remosa" food (highly seasoned or heavy foods)?
A) Yes
B) No
C) I don't know

8. If no, what food?________________________

9. Can the person who was bitten drink liquids?
A) Yes
B) No
C) I don't know

10. If no, which liquid?________________________

11. Which of these objects WOULD YOU USE for snakebite protection? *
J) Flip-flops
K) Long pants
L) Jeans
M) Rubber gloves
N) Boots
O) Flashlight
P) Amulet
Q) I don't know
R) Others: __________

12. What problems/sequelae can an accident cause if the person does not seek help? *
A) Nothing happens
B) The bitten limb might be amputated
C) The person could die
D) The condition could worsen and require hospitalization
E) I don't know
F) Others: __________

Knowledge about snakes

1. Are all snakes venomous?
A) Yes
B) No
C) I don't know

2. Which snake causes the most accidents in the community?
A) Boa constrictor (Jiboia)
B) Anaconda (Sucuri)
C) Spilotes pullatus (Caninana)
D) Jararaca (Bothrops)
E) Bushmaster (Surucucu pico de jaca)
F) Coral snake
G) Rattlesnake

H) I don’t know
