# Supplementary material for: Participatory research towards the control of snakebite envenoming and other illnesses in a riverine community of the Western Brazilian Amazon
Source: PLoS Negl Trop Dis. 2025 Jan 23;19(1):e0012840. doi: 10.1371/journal.pntd.0012840 (PMC11793770; doi:10.1371/journal.pntd.0012840)
Supplement: S3 File — (PDF) [file pntd.0012840.s003.pdf]

INTERVIEW GUIDE – PHASE 1

<table>
<tr><td colspan="2">Researcher's Initial Considerations</td></tr>
<tr><td colspan="2">Hello, good morning (good afternoon), my name is (name), and today I am here to talk a little bit about accidents with snakes. This is not an evaluation, just a conversation to understand your ideas and opinions on this subject. Before we begin, I would like to ask for your consent. There is a document stating that you agree to participate in this conversation. You can read it at your convenience, and I can also explain each item of this document before we start.</td></tr>
<tr><td>Questions/Theme</td><td>Objective</td></tr>
<tr><td colspan="2">Experiences</td></tr>
<tr><td>1. Could you start by introducing yourself?
2. What are the main health problems here in the community? (taking into account the dry season, the flooding, and the period when the river starts to rise)
3. What are the main difficulties regarding access to healthcare here in the community?
4. Have you or any family member ever been bitten by an animal (Where? When?)
5. How was your experience? (From the moment of the accident to the medical attention)
6. How was your medical care? (Understand the entire process)
7. How do you evaluate this care? Why?
8. What was the outcome?
9. In case of death, how was the process? (Understand the whole process in detail</td><td>To know the participant and their personal experiences to foster rapport between the interviewer and the participant.</td></tr>
<tr><td colspan="2">Community scenario</td></tr>
<tr><td>1. How is it when there's a snake accident in the community?
2. Do you always talk about it when you find a snake? (Alerting others)
3. In which season do you think accidents occur more often? (January/February...)</td><td>Understand the scenario of snakebite accidents in the community.</td></tr>
<tr><td colspan="2">Perceptions About the Animal and Risk Activities</td></tr>
<tr><td>1. How do you feel when I say the word "snake"? Why?
2. What do you think people think about snakes?
3. Have you ever heard any stories or superstitions about snakes?</td><td>Understand the patient's perceptions of the animal and what risky activities they may be engaging in for subsistence.</td></tr>
</table>

**4.** What do you feel and what would you do if you found one in the middle of the path or near your house?<br>**5.** Does an accident with a snake mean something to you? (Incapacity, etc.)<br>**6.** Which daily tasks do you think put you most at risk for accidents? Why?<br>**7.** What do you use to protect yourself from accidents while working here in the community? (Apart from snakes, thorns, rocks, vines)<br>**8.** When do people gather and why do they gather? (Times and places)	
Perception of Severity and Seeking Medical Care	
**1.** When do you consider an accident serious? Why?<br>**2.** What's the first thing you would do if bitten by a snake? (Behavior and first aid)<br>**3.** What do people in the community do in these cases?<br>**4.** Could you describe what the first steps of care are?<br>**5.** How would you get to a healthcare unit?<br>**6.** Which healthcare unit would you go to?<br>**7.** Is there anything that would prevent you from seeking medical help? Please describe.<br>**8.** What do you know about the treatment for snake bites?	Understand the perception of the severity of snakebite accidents and the search for medical care. Understand if the participant knows about antivenom and its importance in the first few hours.
Cultural Aspects	
**1.** What forms of care and treatment are done by people here?<br>**2.** Does the community have any "healers"? What do they do?<br>**3.** Why do you think these beliefs are important within the community?<br>**4.** What do you think about home remedies and "city medicine"? (Self-care)<br>**5.** What medicine do you usually prefer and use? (Home-made or pharmacy)<br>**6.** In some places, people tend to walk barefoot. Do you know if that happens here? Could you explain why?<br>**7.** What do you think about this characteristic? (Is it dangerous at times?)	Understand the cultural characteristics to design an appropriate health education intervention.<br>Understand the reasons for the use of traditional medicine and its importance.

**8.** Are there any protective methods used by the locals? (Amulets or charms) What are they?	
Prevention and Use of Personal Protective Equipment (PPE)	
**1.** What measures do you take to prevent accidents? (At home, in the yard...)<br>**2.** How is waste disposed of here in the community?<br>**3.** Is there anything at home that helps you in case of doubts or to prevent diseases?<br>**4.** What do you use to protect yourself when doing activities like farming, fishing, or clearing the land? (Photographing) Why?<br>**5.** When do you not use protective equipment? Why?<br>**6.** Is there anything that would make you not use any of these protective items? Why?<br>**7.** Do you have any suggestions for protective equipment? What would make sense for the community? Describe.	Understand the participant's knowledge regarding prevention methods and identify the methods used in the community. Survey for appropriate personal protective equipment (PPE).
Educational Intervention Inquiry	
**1.** If a serious health problem occurred in the community, what would you do? (e.g., shortness of breath, heart attack, what is the route? How do you communicate? Do you spend any money?)<br>**2.** How would you like this issue to be addressed here in the community?<br>**3.** If I were to conduct an educational session on diseases, how would you like it to be? Why? (Describe in detail)<br>**4.** Do you like leaflets or paper materials?<br>**5.** What works best for you?<br>**6.** Do you have any suggestions?<br>**7.** Is there anything I haven’t asked that you think is important for me to know in order to create the health intervention?	Survey for educational intervention.
Well, Mr./Ms. (name), these were the questions I had for you. If you have any questions, I can answer them now. I would like to thank you for the time you’ve given to talk with our team. All your opinions are very important.	
