# Supplementary material for: Participatory research towards the control of snakebite envenoming and other illnesses in a riverine community of the Western Brazilian Amazon
Source: PLoS Negl Trop Dis. 2025 Jan 23;19(1):e0012840. doi: 10.1371/journal.pntd.0012840 (PMC11793770; doi:10.1371/journal.pntd.0012840)
Supplement: S4 File — (PDF) [file pntd.0012840.s004.pdf]

S4. File. Report prepared at the end of the step 1.

Characterization of Healthcare Access

- ❑ **IDIs, FGD = 32 participants.**

"[...] I think like this: a cough medicine, we can manage to give, because we can have some guidance, right? A medicine for diarrhea, things like that, you know, vomiting, things that are necessary, because it's not every time, if we get a little fever or something, that we're going to a health post. Because sometimes it's hard, sometimes we even get there, and sometimes they don't even see us, right?" (FGD_women).

Distance and Seasonality

"Those who don't have a motorcycle have to paddle the canoe to the other side to get there [...] because, like, during the dry season, he got sick, and we had to paddle the canoe to take him to the hospital." (11EP_PA).

"Full of holes, if someone is sick, with something, they're going to get there dead, because the road is horrible." (FGD_women).

Lack of access medications

Barriers in Seeking Medical Assistance

Monthly Healthcare Visits

"[...] at the beginning of the year, the health agent warned that there would be a vaccine for the week, and the last dose would be on Monday at the UBS. So I went there [...] I made a wasted trip because the right amount wasn't available to open a box of the vaccine." (FGD_Men).

"[...] I've already taken my daughter to do some triage, something with the doctor, and it's always the nurse who does it, the doctor's never there!" (FGD_Men).

Lack of Infrastructure and Human Resources

"Even our health agent, when possible, he takes us." (FGD_women).

"[...] when there are scheduled consultations for people from the community, he chooses the people he will take, like the neighbor next door, or the pastor, or his family members—this is who he chooses to transport there. He doesn't consider the rest of the community [...] The others who go, he says 'if you want, go there,' and he says that to their face without any shame." (11.EP_PA).

Main health problems identified

Contact with animals	Work accidents	Others problems
Scorpions/centipedes	**Accidents with puncture and cutting objects** (hook, machete, glass, steel shards, coins, lumps)	Flu
Catfish		Cough
Spiders/bees		Diarrhea
Caterpillars	**Fractures and sprains**	Viruses
Snakes	Drownings	Low vaccination coverage
Tucandeira ants	Accidents with thorny branches	-
Stingrays	Blows to the body	-

Hook

"[...] he got caught on the hook, and it wasn't just once, it was twice [...] he was about twelve years old [...] the hook got stuck in his leg and went in, and I even did the surgery to remove the hook. I broke a razor blade, right, and cut a little bit [...] I used a lot of strength to remove it, and I managed to take the hook out. So the accident that happened to me, another time, also with him, was with his finger. The hook got stuck in his finger and went through it, but this time I used a bit of intelligence, right, from experience. I passed the hook through, since it had gone through, I passed it through twice, then I hooked the hook onto the canoe and pulled, the hook broke, and I was able to take it out without much effort, but he cried a lot." (05.EP_PA).

"Only once when I was setting the longline, I cut myself. I was cutting and I cut myself, here, cut my knee. I was in a little canoe and I went, and it hit out of nowhere. Despite that, there were no other accidents." (FGD_Men).

Drowning

"I almost drowned once [...] here in front, I was going home, about to cross the river, then I was swimming, and I thought the canoe would hold up, right? I was rowing with paddles because it didn't have a motor yet, then the canoe tipped over, and I went to the bottom. But I had a Styrofoam box in the canoe for the fish, right? So, I went on top of the box and kept shouting in the middle of the river." (FGD_Men).

"Sometimes [the girls?] still don't know how to swim, if they get flooded, right? It's dangerous, because sometimes there are whirlpools, right, and in the whirlpools, the water doesn't have [hair?], right? Where are we going to hold on? Sometimes they drown and drink too much water." (FGD_women).

Animals

"Scorpion - it was right next to the bed, then I didn't see it, I went to put on my shoe and stepped on the floor, on the other side, right where I hadn't put my shoe on, then it stung me. It didn't hurt much, not like a horsefly sting." (FGD_Adolescent).

"The mandim (fish) went through the boot here, in this tendon [...] it entered and broke the spur, this one needed to go to the hospital, but there the doctor didn't do an X-ray, he just used a forceps [...] he searched and didn't find the spur, and after about three months, he went to lie down, and the tip of the spur got caught on the edge of the mosquito net, it hooked. He called his mother, and she came and managed to get it out, I don't know how, and took the spur out. Another time, it was on the arm..." (05.EP_PA).

Other

"Look, here in our region, diarrhea, those flu with cough, fever, it happens a lot when it starts to dry up. There's also been an accident with, here with this... I don't know if you interviewed, uh... stingray here, dragging a canoe, because there's a pipe here, where you all leave, there's a pipe, and he was dragging [pulling] the canoe, and the stingray got him. This happened twice, one here, another time fishing up there." (05.EP).

"Flu is the most... it's what attacks the most, because there was a time it was malaria, but not anymore, now it's this flu that's attacking more." *(*07.EP_PA).
