# Supplementary material for: Participatory research towards the control of snakebite envenoming and other illnesses in a riverine community of the Western Brazilian Amazon
Source: PLoS Negl Trop Dis. 2025 Jan 23;19(1):e0012840. doi: 10.1371/journal.pntd.0012840 (PMC11793770; doi:10.1371/journal.pntd.0012840)
Supplement: S5 File — (PDF) [file pntd.0012840.s005.pdf]

REPORT ON THE COMMUNITY ASSEMBLY

The community assembly was held on September 24, 2022, at the Limeira Municipal School. It included 20 residents, comprising 12 men and 8 women. It is important to note that all 42 participants from the first phase were invited, but due to personal reasons, they could not attend.

The purpose of the assembly was to determine whether the health-related issues identified during the first phase of the study were indeed relevant problems or priorities for the community. These issues were identified after a rapid qualitative analysis of individual interviews (EPs) and focus groups (FGs).

METHODOLOGY

The assembly was based on the culture circles approach: problematization of reality and popular agency. This method promotes a learning process that transcends fragmentation, requiring participants to take a stand on the problems they face. It emphasizes local cultural values and horizontal relationships between educators and learners.

This approach is structured into distinct moments:

- General generative theme: Investigating the vocabulary universe to extract generative words that represent general themes. This is achieved through the educator's “immersion” and “interaction” with the process.
- Thematization: Generative words and themes are coded and decoded to explore their social meanings, allowing participants to broaden their knowledge and understanding of their reality.
- Problematization: Aims to move beyond a naïve perspective toward a critical one, capable of transforming the lived context.

The first two moments of this approach occurred during the initial data collection. From this point, problematization with community members became necessary, which was divided into different steps:

- Initial Moment: Contextualization and presentation of the themes.
- Assembly Moment: Participants were divided into pairs to discuss the chosen themes, add suggestions, share information, and propose solutions. Each pair then presented

their themes and suggestions to the group, allowing others to contribute additional information.

- Priority Definition Moment: At the end, researchers explained that the themes needed to be organized in order of priority. Participants were given time to discuss and reach a consensus. During this step, researchers stepped out of the assembly environment.

RESULTS

During the presentation of the themes, a brainstorming session was conducted for all discussed topics. Below are some of the additional points raised:

- **Accidents caused by venomous animals**: Occur during leisure activities; annual monitoring by a biomedical team suggested; need for someone trained with medication; lack of health access during the dry season; need for road improvements; suggestion for a basic health unit with a nurse or technician at a minimum.
- **Women's health**: Need for trained personnel to administer contraceptives; delays in preventive exams and results; private healthcare as a faster alternative; delays in mammograms in the region; lack of service at basic health units; need for health rights education.
- **Viruses**: Severe body pain affecting children and the elderly; solutions include training in first aid, access to medication, and transportation to health units; need for vehicles suitable for dry and flood seasons; water treatment education.
- **Accidents involving sharp objects**: Occur due to nails, needles, wood pieces, debris from the river, and gardening; suggestions include wearing long pants and careful yard maintenance.
- **Fractures and sprains**: Often due to slips or head injuries; suggested solutions include picking fruit with a basket attached to a pole, first-aid training, and using a motorcart for hospital transport during the dry season.
- **Drownings**: Primarily affect children near the river while swimming; suggestions include explaining water risks during storms, floods, and collapses caused by falling trees.

- **Domestic accidents**: Involve hot oil, sugar, kerosene, hot water, outboard motors, pressure cookers, and frying fish; traditional remedies like cashew resin or oils are used; suggestions include burn severity education and providing ointments.
- **STIs**: Affect adults, particularly the elderly; proposed solutions include condom distribution and general education on safe sex and sexual health.
- **Seizures**: Suggestions include organizing lectures by specialists, creating educational leaflets, ensuring technical health assistance, using illustrative materials, and training a rescue team in the community.

PRIORITY ORDER

The order of priorities selected by participants was as follows:

1. Accidents caused by venomous animals
2. Women’s health
3. Viruses
4. Accidents involving sharp objects
5. Fractures and sprains
6. Drownings
7. Domestic accidents
8. STIs
9. Seizures
