# Supplementary material for: Participatory research towards the control of snakebite envenoming and other illnesses in a riverine community of the Western Brazilian Amazon
Source: PLoS Negl Trop Dis. 2025 Jan 23;19(1):e0012840. doi: 10.1371/journal.pntd.0012840 (PMC11793770; doi:10.1371/journal.pntd.0012840)
Supplement: S6 File — (PDF) [file pntd.0012840.s006.pdf]

CODEBOOK

Riverine community_Alicia.mx20

02/12/24

Code List

Codes and subcodes	Nº of coded segments
**Snakebite in the community**	**414**
Describing contact/accidents with snakes	137
Describing knowledge about snakes	129
Relating to the animal	123
Perceiving venom effects	25
**Accidents with other venomous animals**	**144**
Describing contact/relationship with bats	3
Describing experience with fish/stingrays	47
Describing experience with spiders/scorpions	78
Perceiving scorpions/centipedes	16
**Other health problems faced**	**491**
Accidents during daily activities and work	178
Serious health issues/diseases	83
Women's health	56
Health problems during the dry season	56
Problems during the flood season	59
Drownings	4
Describing other diseases	55
**Challenges faced in accessing healthcare**	**365**
Searching for places for medical assistance	77
Barriers faced in accessing healthcare	131
Feeling on your own	42
Feeling invisible/excluded/abandoned	34
Arriving at medical care/hospital	81
**Improvements in healthcare access**	**234**
Suggesting improvements for healthcare access	42
Talking about educational actions/proposals	141
Topics	51
