# Supplementary material for: Participatory research towards the control of snakebite envenoming and other illnesses in a riverine community of the Western Brazilian Amazon
Source: PLoS Negl Trop Dis. 2025 Jan 23;19(1):e0012840. doi: 10.1371/journal.pntd.0012840 (PMC11793770; doi:10.1371/journal.pntd.0012840)
Supplement: S7 File — (PDF) [file pntd.0012840.s007.pdf]

Consolidated criteria for reporting qualitative studies (COREQ): 32-item checklist - Participatory research towards the control of snakebite envenoming and other illnesses in a riverine community of the Western Brazilian Amazon (1).

No. Item	Guide questions/description	Reported on Page #
**Domain 1: Research team and reflexivity**		
Personal Characteristics		
1. Inter viewer/facilitator	*Which author/s conducted the interview or focus group?*<br><br>APCS, EARTS, and HSSG.	Community baseline assessment, 13 and 14.
2. Credentials	*What were the researchers' credentials?*<br><br>PhD, MD.	Research team and reflexivity, 11 and 12.
3. Occupation	*What was their occupation at the time of the study?*<br><br>University professors, and qualitative researchers.	Title page.
4. Gender	*Was the researcher male or female?*<br><br>Seven women and six men.	Research team and reflexivity, 11 and 12.
5. Experience and training	*What experience or training did the researcher have?*<br><br>Renowned researchers, as well as graduate students with experience in qualitative research, all with qualitative research training and with published articles in the area.	Community baseline assessment, 11 and 12.
Relationship with participants		
6. Relationship established	*Was a relationship established prior to study commencement?*<br><br>None of the participants had an established relationship with an author prior to study commencement.	Research team and reflexivity, 12.
7. Participant knowledge of the interviewer	*What did the participants know about the researcher? (e.g., personal goals, reasons for doing the research).*<br><br>Participants were briefed on the purpose of the study. Educational ethical approval had been granted and participants reviewed the participant information documentation prior to giving their written informed consent to be involved.	Ethics statement, 18.

8. Interviewer characteristics	*What characteristics were reported about the inter viewer/facilitator? e.g., bias, assumptions, reasons and interests in the research topic.*<br><br>No interviewer-related biases were identified.	Research team and reflexivity, 11.
**Domain 2: study design**		
Theoretical framework		
9. Methodological orientation and Theory	*What methodological orientation was stated to underpin the study? e.g., grounded theory, discourse analysis, ethnography, phenomenology, content analysis.*<br><br>Qualitative data analysis and interpretation occurred simultaneously in Step 1 of the study through rapid qualitative analysis, utilizing debriefings.<br><br>Braun and Clarke's thematic analysis was conducted using MAXQDA 20 software in step 3.	Community baseline assessment, 15 and 16.<br><br>Final analysis, 17 and 18.
Participant selection		
10. Sampling	*How were participants selected? e.g., purposive, convenience, consecutive, snowball.*<br><br>The study employed a multi-method sampling strategy tailored to each research method.	Sampling approach, 12 and 13.
11. Method of approach	*How were participants approached? e.g., face-to-face, telephone, mail, email.*<br><br>We did a face-to-face.	Community baseline assessment, 14 and 15.
12. Sample size	*How many participants were in the study?*<br><br>42 people	Sampling approach, 12 and 13.
13. Non-participation	*How many people refused to participate or dropped out? Reasons?*	N/A
	No one has given up on participating in the project.	
Setting		
14. Setting of data collection	*Where was the data collected? e.g., home, clinic, workplace.*<br><br>The IDIs were carried out at homes and FGDs were carried out at school.	Community baseline assessment, 15.

15. Presence of non-participants	*Was anyone else present besides the participants and researchers?*<br><br>No, only the interviewer, the observer and the participant were in the room during the interview.	Community baseline assessment, 15.
16. Description of sample	*What are the important characteristics of the sample? e.g. demographic data, date*<br><br>Study area and population characteristics.	Study area and population characteristics, 8.
Data collection		
17. Interview guide	*Were questions, prompts, guides provided by the authors? Was it pilot tested?*<br><br>A semi-structured interview guide with open-ended questions was developed by the research team, who have expertise in qualitative studies, and was validated with a smaller sample of volunteers	Community baseline assessment, 14.
18. Repeat interviews	*Were repeat inter views carried out? If yes, how many?*<br>*No.*	N/A
19. Audio/visual recording	*Did the research use audio or visual recording to collect the data?*<br><br>The interviews were recorded in audio and transcribed without personal identifiers, so that the database could be anonymized.	Community baseline assessment, 15.
20. Field notes	*Were field notes made during and/or after the inter view or focus group?*<br><br>The observer took field notes in step 1.<br>The researchers took field notes in step 2.	Community baseline assessment, 15.<br><br>Community assembly, 17.
21. Duration	*What was the duration of the interviews or focus group?*<br><br>The IDIs lasted an average of 60 minutes, while the FGDs lasted 80 minutes.	Community baseline assessment, 15.
22. Data saturation	*Was data saturation discussed?*<br><br>The number of interviews was determined by the principle of theoretical saturation where IDIs are carried out until a clear pattern appears and subsequent groups do not produce new information.	Community baseline assessment, 13.
23. Transcripts returned	*Were transcripts returned to participants for comment and/or correction?*<br><br>No.	N/A

**Domain 3: analysis and findings**		
Data analysis		
24. Number of data coders	*How many data coders coded the data?*<br><br>The data were coded by APCS, and the preliminary codebook was reviewed by FLGM and developed the final codebook.	Final analysis, 15.
25. Description of the coding tree	*Did authors provide a description of the coding tree?*<br><br>No.	N/A
26. Derivation of themes	*Were themes identified in advance or derived from the data?*<br><br>The analysis of the IDIs, FGDs, field notes and community assembly allowed us to identify the four major themes.	Final analysis, 17 and 18.
27. Software	*What software, if applicable, was used to manage the data?*<br><br>The recordings of the IDIs and FDGs were transcribed and inserted in the MAXQDA 20 software.	Final analysis, 18.
28. Participant checking	*Did participants provide feedback on the findings?*<br><br>No.	N/A
Reporting		
29. Quotations presented	*Were participant quotations presented to illustrate the themes/findings? Was each quotation identified? e.g. participant number*<br><br>Yes, quotations were presented to illustrate the themes/findings, and each quotation was identified with a participant number, gender, years old and occupation.	Results, 22-33.
30. Data and findings consistent	*Was there consistency between the data presented and the findings?*<br><br>Yes, there was consistency between the data presented and the findings.	Discussion, 33-38.
31. Clarity of major themes	*Were major themes clearly presented in the findings?*<br><br>Yes, major themes were clearly presented in the Results section using specific sections regarding each theme.	Results, 22-33.

32. Clarity of minor themes	*Is there a description of diverse cases or discussion of minor themes?*<br><br>No, minor themes were not discussed.	N/A

REFERENCES

1- Allison Tong, Peter Sainsbury, Jonathan Craig, Consolidated criteria for reporting qualitative research (COREQ): a 32-item checklist for interviews and focus groups, International Journal for Quality in Health Care, Volume 19, Issue 6, December 2007, Pages 349–357, https://doi.org/10.1093/intqhc/mzm042
