# Supplementary material for: Participatory research towards the control of snakebite envenoming and other illnesses in a riverine community of the Western Brazilian Amazon
Source: PLoS Negl Trop Dis. 2025 Jan 23;19(1):e0012840. doi: 10.1371/journal.pntd.0012840 (PMC11793770; doi:10.1371/journal.pntd.0012840)
Supplement: Attachment — Submitted filename: Response to Reviewers.docx [file pntd.0012840.s008.docx]

**Response to Reviewers**

Reviewers' Comments:

**Reviewer #1**

The objective of this study is clear, aiming to identify and address health issues faced by the Limeira community in the Brazilian Amazon basin through participatory research, particularly focusing on snakebite envenomation (SBE) and other common health problems. However, despite the clarity of the goal, the study does not present a clear testable hypothesis. This raises a question: in such a complex social and health context, should hypotheses be more explicitly defined to guide the research direction, especially when dealing with numerous unknown health challenges? I suggest that the authors provide a broader social context in the 'Introduction' section, rather than only discussing the Amazon region.

**Answer:** We have revised the Introduction to provide a broader social context and added a clear hypothesis to guide the study (Line 75-87 and 124-128).

**_______________________**

**Reviewer #2**

(240): Purposive sampling is a sound approach to this type of study. Further explanation of questionnaire design and the purpose of each question would be helpful. Research design is sound, with appropriate methods used to construct and test the SSI instrument.

**Answer:** We have included details on the design and purpose of the semi-structured interview guide to address this point (line 480-485).

(270): What type of recordings? Audio? Video?

**Answer:** We agree with the revisor, and that information was added (line 490 and 501).

**(**275): Focus groups typically consist of a dominant speaker who controls the conversation, thus essentially resulting in the equivalent of one SSI and diminishing the sample size.

**Answer:** You are correct, but we believe that in our study, focus groups, moderated to ensure balanced participation, effectively complemented individual interviews and participant observations, providing more reliable findings.

(308) - Was cultural consensus analysis à la Romney, Weller and Batchelder ever calculated from the data? This result could then be compared with the outcome of Freire’s consensus building method results.

**Answer:** We didn’t apply cultural consensus analysis in this study, as our methodological approach focused on participatory methods inspired by Paulo Freire’s approach.

(376) – Was the ranking task only conducted at the focus group level? It would be interesting to compare group results with those of individuals as a collective as a validity test.

**Answer:** The ranking task was conducted collectively during the community assembly, where participants prioritized health issues as a group. This activity was not replicated at the individual level, as the focus of our participatory approach was on fostering collective discussion and consensus. While comparing group and individual results could provide additional insights, we did not pursue this at that time.

(416) – The information on the number of interviews and how focus groups were determined should come sooner. While it is not stated, it appears the researchers were interested in comparing gender and age as variable; if so, this should be stated outright. (There is no discussion of differences of variables vis-à-vis FGs anywhere in the paper).

**Answer:** Thank you for your comment. We did not intend to compare gender; the separation was made solely to ensure that participants felt more comfortable during the interviews.

(429) – Need a line or two stating that themes are being presented drawn from either just the SSIs, or both the SSIs and FGs.

**Answer:** That information was added (line 706-708).

(461) - On page 400, It is presented that 50% of residents report walking barefoot, but on 461 it states that participants said SBE is rare due to people wearing protective gear. This discrepancy may be an artefact of the different data collection methods. Some explanation of this discrepancy, especially the possible reason for it, is warranted.

**Answer:** The finding that 50% of residents reported walking barefoot reflects general practices in their daily lives, often near their homes or in less SBE risky situations. In contrast, participants' statements about SBE being rare due to protective gear refer specifically to their precautions during activities perceived as high-risk, such as farming. We will clarify this distinction in the text to provide better context for these findings (line 668-670).

(537)- If other common health issues are to be included, they should be directly related to SBE, and the purpose of their inclusion stated – for example, lack of treatment of other health problems correlating with lack of treatment of SBE, etc. (Table 5 makes it seem that there is not a great deal of reasons for including these other health problems, and they appear somewhat superfluous to the study).

**Answer:** We understand your concern; however, the inclusion of other common health issues, such as women's health, viral illnesses, and accidents, was integral to the participatory approach of this study. During the community assembly, participants highlighted these problems as interlinked with the broader challenges of accessing healthcare services in the region. For example, the same barriers preventing timely treatment for SBE, such as lack of infrastructure and limited transportation, also hinder access to care for these other issues. The text has been revised to better reflect this rationale (line 998-1002).

(564) – This entire section on other health problems detracts from the primary focus of your research. A focus on SBE here, with more detailed information, would better serve the purpose of the paper. Side data such as this is often collected, which could be another paper, or could be the groundwork for further studies, but a brief mention, rather than an entire section would be better suited.

**Answer:** The objective of this study is to identify the health problems faced by riverine communities and to understand SBE within this broader context. Excluding these issues would overlook their relevance and contradict the collaborative analysis conducted with the community, which emphasized the interconnected nature of these health challenges. As noted previously, we have adjusted the text to better align with this perspective and maintain the study's focus

**_______________________**

**Reviewer #3: INTRODUCTION**

(1) Structure (lines 74-138): This study sought to deepen understanding on the role of SBE and other health challenges in a riverine community and identify strategies to address these challenges. The Introduction focuses on the historic, political, and economic factors influencing health of people in riverine communities. Whilst this background is important, especially for readers not familiar with this context, the problem of SBE, which this sought to address, should be stated upfront (ideally in the 1st paragraph). The contextual background can follow. In order to make a stronger case for the study, authors could state the evidence gap (why is this study needed) more clearly.

**Answer:** We have restructured the Introduction as suggested (line 75 – 87).

**METHODS**

Need for more concise presentation of methods and the translation of theory into methods and activities.

(2) General comment: Kindly edit to reduce unnecessary repetitions (including but not limited to the steps, methods, theory underpinning community assembly etc.)

**Answer:** We agree with the reviewer and have reduced unnecessary repetitions. However, some details were retained to ensure clarity and preserve the comprehensive understanding of the methodology.

(3) Ethics Statement (lines 141-147): Whilst this sub-section is important, it may be more effective to begin the Methods section with the context of the study (lines 175-182) and move ethics to the end of Methods. Please include a reference to supplementary ‘COREQ’ checklist. Authors may also outline measures put in place to ensure ethical conduct of the study.

**Answer:** We have restructured the Methods section as suggested. The Ethics Statement has been moved to the end of the Methods section (lines 608-618), and the context of the study is now presented at the beginning (lines 159-272). A reference to the supplementary 'COREQ' checklist and the ethical measures taken during the study have also been included.

(4) Study design (lines 184-218 and 247-354): (a) According to Methods, this study was proposed and designed by the research team and used a mixed-methods research design involving quantitative, qualitative, and participatory methods. The principles of Paulo Freire’s participatory education served as the epistemological foundation for the community assembly, not the entire study. Although authors state participation and horizontal power relations in theory, the Methods do not clarify how these were implemented in practice at the different stages of the research, except for the community assembly. There is need for more critical reflection on the level of participation of community members in the study beyond providing data (e.g., development of questionnaires and topic guides, enumerators and facilitators, data analysis). For example, how did the authors achieve a horizontal educator-learner relationship, when they designed the study, collected and analyzed data using software (step 1), and began the community assembly (step 2) with a presentation of their results from step 1?

**Answer:** The Horizontal relationships were primarily established during the community assembly. After the initial analysis, participants validated the findings presented by the researchers, adding relevant information and removing those they deemed unnecessary. In this way, they actively contributed to the data analysis process, reinforcing the collaborative nature of the research (354-357).

(b) Kindly state the duration of the study.

**Answer:** We added this information as suggested (line 261-262).

(5) Building rapport with the community (lines 220-229): (a) This sub-section outlines the main engagements with the community – introduction of the project, data collection, and validation (Community Assembly) – beyond building rapport. Instead of having a separate sub-section, authors may include the first two visits to the community as Step 1 “building rapport with the community” in their study design, whilst also providing more detail what happened during these visits. The other visits are already covered in study design (lines 184-218), removing these will help reduce duplication and word count.

**Answer:** That information was changed as suggested (line 447-465).

(b) There are many abbreviations in this sub-section. Readers are not familiar with these abbreviations. Please introduce all of them when using them the first time.

**Answer:** We used abbreviations initially to simplify the text, but we acknowledge the suggestion and have made the necessary changes. All abbreviations are now introduced upon first use (lines 377–407).

(6) Research team and reflexivity (lines 231-238): Kindly consider using initials to explain the positionality of the different researchers. Please explain which efforts were made to “prevent their personal perspectives from directly influencing data collection and analysis.”

**Answer:** That information was added (line 374-409).

(7) Sampling (lines 240-246): (a) The study involved different methods – cross-sectional survey (structured questionnaire), in-depth interviews, focus group discussions, participant observation, and community assembly. Authors recruited “individuals who can provide valuable insights into the research topic.” Sampling and recruitment should be reported for each method separately, including the number and characteristics of participants recruited.

**Answer:** The information was added (line 411-443).

Study area (lines 148-173): The description of the seasons affecting people in this context. The size of the population and main economic activities (lines 358-359) may be reported in this sub-section, instead of Results, and provide more details like age profile, gender composition if available.

**Answer:** We agree with the revisor, and that information was added (line 273-325).

(b) The study involved minors, please clarify the age of maturity and explain the consent procedures for minors. Please state who facilitated informed consent procedures (e.g., initials).

**Answer:** The information about the inclusion of minors, specifying the age of maturity, and the consent procedures for minors has been added (lines 252–253).

(8) Step 2 (lines 306-332): The structure of the section is difficult to comprehend because of a separation between the theory of the method (lines 319-323) and how it was implemented (lines 323-328), using different terminology. The theory part (lines 308-319) could be shortened.

**Answer:** The section has been revised for clarity (line 534-544).

**_______________________**

**REVIEW: RESULTS**

**Reviewer #1**

I am not an expert in participatory research, So I recommend inviting an additional reviewer with more specialized knowledge to offer professional insights. In section 'Discussions', I propose the authors should discuss how their study be spread in other similar regions. Replicability and scalability may be influenced by different cultural backgrounds and geographic conditions, and these aspects should be discussed deeply.

**Answer:** We have added to the Limitations section that this type of study is not replicable due to the unique cultural and geographical contexts. However, we emphasized that it can provide valuable insights into understanding barriers and facilitators in similar settings, highlighting how qualitative research can contribute to the development of tailored interventions (line 1062-1079).

**_______________________**

**Reviewer #2**

It would be helpful if the codebook were included as an appendix, as well as MAXQDA outputs such as matrices, frequencies, etc.

**Answer:** We agree with the revisor, and that information was included as a supplementary S6 file (line 602).

Table 1 – Were all 42 participants involved in each of the data collection methods, i.e., questionnaires, SSIs, focus groups? For example, were there 42 questionnaires, 42 SSIs, and how many focus groups?

**Answer:** The information regarding participant involvement in each data collection method has been clarified and improved (lines 255-257).

**_______________________**

**Reviewer #3**

Need for better integration of quantitative and qualitative findings.

(9) Participant characteristics (lines 357-373 and lines 416-423): Authors describe the quantitative sample of 42 individuals in greater depth than the qualitative sample. More detail about in-depth interview, focus group discussion, and community assembly samples needed; the labels of quotes suggest such information were collected. The participant observation sample is missing; please add.

**Answer:** We agree with the revisor, and that information was improved (line 401-433).

Consider reporting participant characteristics in Methods (sampling) to avoid breaking up Results.

**Answer:** We agree with the revisor, and that information was improved (line 299-314).

(10) Main health problems (lines 375-414 and lines 537-562): The presentation of common health problems in the community is disjointed and, in addition, separated by the SBE findings (Theme 1). The quantitative results “Identification of the main health problems” and qualitative findings “Theme 2: Common health issues in the community” should be integrated and presented as one area of results, providing a context in which the community encounters SBEs. In addition, have authors thought to disaggregate the responses by age, gender, or other relevant characteristics as health issues appear to be related to domestic, occupational activities among adults and play among children (Table 4)?

(11) SBEs (lines 385-414 and lines 428- 535): Similarly, the quantitative evidence on exposure to venomous animals (lines 385-414) and qualitative findings on experience of SBEs (Theme 1, lines 428- 535) should be integrated and presented as one result area.

**Answer:** We chose to present quantitative and qualitative findings separately to maintain methodological clarity and depth. The study design did not include sufficient stratification by age or gender for robust analysis, as the focus was on general health challenges and their intersection with SBE.

(12) Theme 3 (lines 564-639): Consider adding sub-headings for the sub-themes. It is surprising to see that participants proposed solutions are mainly curative in response to ill-health and less often preventive, except for condom use.

**Answer:** We agree with the revisor, and that information was added (line 862 and 889).

**_______________________**

**REVIEW: CONCLUSIONS**

**Reviewer #1**: The manuscript uses a qualitative analysis method. Although the authors also provide some descriptive statistics based on previous surveys, overall, it is insufficient. I recommend the authors provide more supporting data as appendixes to ensure the scientific research. This includes participant information, ethical approve, questionaire, etc. Regarding limitations, the authors should discussion the limitations at the last paragraph of the section 'Discussions', including the issues encountered during sample selection and data analysis. Additionally, targeted and actionable suggestions should be offered as references for similar research. This study aligns with the sustainable development goals proposed by UNESCO.

**Answer:** As per the suggestions of Reviewer 2 and your recommendations, we have included additional information in this revised version, such as the codebook, and improved the ethics section to provide more comprehensive details. We also added a clearer explanation of the questionnaire, including how it was designed and validated. Furthermore, following your suggestion, we improved the study's limitations in the last paragraph of the discussion section, addressing issues related line 1062-1079).

**_______________________**

**Reviewer #2:** 642 – The socioeconomic findings should be presented earlier at the beginning of your data findings since this data provides context for your health findings. It does not belong in the discussion section, other than as a framing.

**Answer:** The text has been revised as suggested, with the socioeconomic findings moved to the beginning of the data findings section (line 309-325).

Again, the superfluous health data in the discussion section seems unnecessary and inappropriate.

**Answer:** We appreciate the feedback. Regarding the comment on the health data in the discussion section, we believe its inclusion is essential to contextualize the findings, enhancing the understanding of the challenges faced by the community and aligning with the participatory approach of the study. Moreover, highlighting multiple health issues is crucial to contextualize snakebite envenoming within a broader perspective and advocate for integrated solutions.

656-664 – This ten-year reduction in SBEs is a very interesting finding. I wish there was more discussion of this change in cultural knowledge, especially implemented into the research design, with clearer data analysis of how and why this change occurred within this specific community.

**Answer:** We have incorporated modifications in the discussion section to address the observed ten-year reduction in snakebite cases. This includes an expanded discussion on cultural changes, such as increased accessibility to protective equipment due to price reductions in the past five years, and their role in reducing incidents (line 932-963).

I am disappointed that there is not more data collection and analysis presented on the barriers to resolving SBEs within the community. Participants provide solutions, but the likelihood of these solutions being implemented by the government are not adequately discussed. It would have been helpful if this study included all stakeholders, therefore providing better value.

**Answer:** Thank you for your feedback. We understand the importance of including all stakeholders to provide a more comprehensive analysis. However, due to the scope and resources of this study, our focus was limited to the community perspective. While the participants' solutions were highlighted, a deeper engagement with government representatives and other stakeholders was beyond the current study's capacity. We agree that this is a critical area for future research and appreciate your suggestion, which will guide our next steps in expanding the scope of this work.

742 – I am not left with the impression that “this study provides valuable insights” other than the 10-year change in cultural knowledge and practice. Please go into detail about the specific valuable insights.

**Answer:** We have rewrite this conclusion to make it more aligned with the findings of the study (1082-1092).

753 – Decentralizing is important, but how would that work for this community specifically?

**Answer:** We have removed this phrase as per your suggestion to make the text more consistent with the findings of the study.

758 – I agree that community participation is a necessity, but those in control of the government apparatus need to hear the community directly, and require intermediaries since marginalized people are at a power disadvantage. This study would have been a perfect opportunity to mediate between community, health officials, and government officials, while advocating for the community.

**Answer:** We fully agree with your comment. Mediating between the community, health officials, and government representatives is essential. Future initiatives by our study group will prioritize fostering this dialogue and advocating for the community.

**_______________________**

**Reviewer #3: DISCUSSION**

Need for greater focus on answering the main research question.

(13) Main findings (lines 643-653): Authors begin the Discussion with a summary of participants’ sociodemographic data. However, it may be more effective to link this paragraph on the research question, providing a summary of key findings in response to the main and specific objectives. In addition, there is a disconnect between the sub-heading “River’s identity, subsistence, and invisibility” and the content; this paragraph may not need a sub-heading at all.

**Answer:** We have restructured the discussion to better align with the main research question and objectives. The section now begins with a focus on the key findings related to SBE and its place within the community's reality. Also, the subheading “River’s identity, subsistence, and invisibility” has been removed to improve clarity and coherence (line 931-963).

(14) Coherence: Reports of the importance of SBE in the riverine community are inconsistent. On the one hand, accidents involving venomous animals are perceived as a severe health problem (line 430) and ranked as the priority number one health issue in the community (Table 2). On the other hand, participants reported SBEs to be rare events (line 461), with the reported cases dating back over 10 years (lines 656-657). A discussion of these contradictory results is needed, including the potential of social desirability bias, given authors’ interest in SBEs.

**Answer:** We agree with your observation and have addressed this point by incorporating the information into the last paragraph of the discussion, where we discuss it as a limitation of the study (line 1062-1079).

**CONCLUSION**

(15) Conclusion (lines 742-747): The messages in the Conclusion should be focused on the research aim and data. The study generated evidence on the diversity and priorities of health issues among the riverine community as well as their proposed solutions to achieve good health. The summary of evidence in Conclusion may be misleading since “health conditions of a riverine community in the Brazilian Amazon” and “barriers to healthcare access” are not within the scope of the study.

**Answer:** We have rewritten the conclusion to align more closely with the research aim and findings (line 1081-1097).

(16) In addition, authors highlight the importance of “participatory research”, “community participation in project development”, “interdisciplinary approach”, and “methods that promote the agency of riverine communities”. Whilst not disputing the importance of participatory methods for health policy and programming, this study was not designed to assess the effectiveness or meaning of such methods. Authors may consider a critical reflection on methods in a separate manuscript if such data were available.

**Answer:** Thank you for your observation. We acknowledge that this study was not designed to assess the effectiveness of participatory methods and agree that a critical reflection on these methods could be explored in a separate manuscript.

(17) Authors promote “invisibility” as a theme in the Title, Introduction, Discussion, and Conclusion of the manuscript, whilst “invisibility” does not explicitly feature as a theme in Results. This is not to dispute that the health needs and health concerns of the riverine population may be invisible in policy and programmes, generating the key message inductively from the data can help strengthen the manuscript. Authors may consider changing the title, dropping “Turning the invisible into visible”, and, instead, highlight participants’ request for structural, capacity, and medical support to be able to better manage their health and a health system strengthening approach to addressing SBE and other health issues.

**Answer:** We agree with your suggestion and have removed "Turning the invisible into visible" from the title (line 3).

**_______________________**

**Editorial and Data Presentation Modifications?**

Use this section for editorial suggestions as well as relatively minor modifications of existing data that would enhance clarity. If the only modifications needed are minor and/or editorial, you may wish to recommend “Minor Revision” or “Accept”.

**Reviewer #1:** Based on the aforementioned suggestions, I recommend a major revision. The authors should address the issues outlined above appropriately.

**_______________________**

**Reviewer #2:** 135: Sentence on study objective suffers from clarity issues.

**Answer:** We have revised the sentence on the study objective to improve clarity and ensure it accurately reflects the focus and scope of the manuscript (line 124-128)

144: Participants “were” provided a sheet of information on the study (?)

**Answer:** We agree with the revisor, and that information was added (line 613-615).

Were participants all literate?

**Answer:** We revised the ethics section according to your suggestion to provide greater clarity regarding the process of obtaining consent, especially for the four illiterate participants.

220: The section on rapport building is rather anemic. Only the first visit was solely for building rapport, the others were data collection. What value did the researchers bring to the community on the first visit to build rapport?

**Answer:** We revised the section to clarify the value brought to the community during the first visit (line 437-455).

**_______________________**

**Reviewer #3: Abstract**

Please separate Methods and Results (two paragraphs). Kindly state the duration of the study, number of participants, and data collection methods. More details on the Results and less emphasis on Background and Conclusions.

**Answer:** We agree with the revisor, and that information was changed (line 35-51).

**_______________________**

**Summary and General Comments**

Use this section to provide overall comments, discuss strengths/weaknesses of the study, novelty, significance, general execution and scholarship. You may also include additional comments for the author, including concerns about dual publication, research ethics, or publication ethics. If requesting major revision, please articulate the new experiments that are needed.

**Reviewer #1:** This study focuses on the issue of snake bites in remote regions of the Amazon, addressing a neglected public health problem. Given the high incidence of snake bites in this area, the manuscript holds significant importance in the field of neglected tropical diseases. The solutions proposed in the study, such as first aid training, access to satellite phones, and infrastructure improvements, are practical and can directly inform local health policy interventions. However, I believe the authors still need to address the following issues to enhance their chances of publication.

Existing Issues:

1. It is recommended that the authors conduct a more in-depth discussion of the descriptive statistics of the questionnaires and interviewees. This should include how differences in occupation, age, gender, and culture affect attitudes toward health issues, which would help improve the design of community services.

**Answer:** We have improved the text by addressing how occupation, age, gender, and cultural beliefs influence attitudes toward health issues (line 673-684).

2. As mentioned above, based on the study results, can the authors establish several priority actions to illustrate how the findings can be extended to other tropical communities?

**Answer:** We have revised parts of the discussion and conclusion to address this suggestion (line 1088-1092).

3. Additionally, why did the authors choose subjective sampling instead of stratified or mixed sampling? The authors need to provide sufficient justification for this choice, as it directly affects subsequent research results. For example, subjective sampling may exclude more marginalized groups in the community or those less familiar with the health system.

**Answer:** We have added a justification for the use of convenience sampling in the methodology (line 417-424).

**_______________________**

**Suggestions to authors:**

1. Enhance the Depth of Quantitative Analysis:In the subsection "Step 1 - Community baseline assessment," page 11, third paragraph, only simple descriptive statistical analysis of the collected questionnaire data is currently presented. It is recommended to add further quantitative analysis after this paragraph, using multivariate analysis methods (such as linear regression or logistic regression) to explore the correlation between different demographic characteristics (e.g., gender, age, occupation) and various health issues. This will make the conclusions more reliable and facilitate the generalization of results to other similar communities.

**Answer:** Thank you for the suggestion. However, this study primarily employs a qualitative approach, with quantitative data serving to complement some of the qualitative findings. Given the use of convenience sampling, the focus was on capturing diverse perspectives rather than ensuring statistical representativeness, making descriptive statistics more appropriate for our objectives.

2. It is suggested to add a follow-up plan based on the existing research framework to observe the long-term effects of the proposed interventions (such as first aid training and infrastructure improvements). By collecting data on changes in community health status before and after the interventions, the actual impact of these measures can be better evaluated, thus providing stronger evidence for future public health policies.

**Answer:** Thank you for the suggestion. We are planning a follow-up study, starting in 2025, to evaluate the long-term effects of the proposed interventions and their impact on community health.

3. Expand the Discussion on Environmental Factors: In the "Discussion" section, page 34, second paragraph (the paragraph starting with "The frequency of snake encounters is high"), after discussing the frequency of community members' contact with venomous snakes, it is recommended to further elaborate on the impact of climate change and environmental degradation on snake behavior and other health issues. Relevant studies can be cited to illustrate how climate change increases the range of snake activity, thereby raising the risk of snake bites. Additionally, other environmental issues (such as deforestation and deterioration of river water quality) and their potential impacts on infectious and occupational diseases can be discussed. This can elevate the study's discussion to the context of global environmental changes, enhancing the paper's cutting-edge nature.

**Answer:** Thank you for the suggestion. We have added a discussion on how climate change and environmental degradation impact snake behavior and health risks, supported by relevant studies (Line 973-994).

**_______________________**

**Reviewer #2:** This study provides value as a case study, especially in terms of the 10-year difference observed in SBE cultural knowledge and adaptation. I would recommend shifting the framing of the writing to highlight this finding. As outlined, I found the data on other health problems to be distracting from the focus of the study.

**Answer:** We have made several adjustments to the text to clarify that addressing other health problems supports community engagement efforts for SBE.

**_______________________**

**Reviewer #3:** Researchers proposed this study to establish the importance of and solutions to snake bite envenoming among riverine communities in Brazil, using a mixed-methods research design involving quantitative, qualitative, and participatory methods. They engaged community members on several occasions during the study to build rapport, collect data, and develop solutions. Researchers made an effort to engage with the views of participants from the community, including widening the research focus to include other conditions, but evidence for their consistent involvement in horizontal power relations is limited. The Results provide opportunities for action. However, their presentation and discussion may be integrated and focused on the research question. Given the active role researchers have in a study like this, active language and author initials may allow to reader to better understand who carried out what activity. This review identifies opportunities to strengthen this manuscript like making a stronger case for the study (Introduction); presenting methods more concisely, including how theory was translated into methods and activities (Methods); integrating quantitative and qualitative findings (Results); and strengthening the Discussion to focus on the research question (Discussion/Conclusion).

**Answer:** Thank you for your feedback. We have revised the text to integrate the results and discussion and to clarify the researchers' active role, addressing your suggestions.
