# Supplementary material for: Participatory research towards the control of snakebite envenoming and other illnesses in a riverine community of the Western Brazilian Amazon
Source: PLoS Negl Trop Dis. 2025 Jan 23;19(1):e0012840. doi: 10.1371/journal.pntd.0012840 (PMC11793770; doi:10.1371/journal.pntd.0012840)
Supplement: Attachment — Submitted filename: Response to Reviewers.docx [file pntd.0012840.s009.docx]

**Response to Reviewers**

**Methods**

**Reviewer #1:** Issues on old manuscripts have been resolved.

**Reviewer #3:** This version of the manuscript is clearer, adding suggestions for minor corrections.
**Introduction:** The flow is improved. However, the added hypothesis seems to focus on the effectiveness or value of a participatory research approach, not the views of a riverine community regarding their health issues and healthcare needs. I would advise against mixing methodological and empirical findings. Authors may consider evaluating the methodological approach, offering a critical reflection in a separate manuscript.

**Answer:** We have revised the text accordingly (lines 136–142).

**Methods:** Information requested has been added and methods clarified.
Sampling: It seems a stratified sampling approach was used for FGDs. The sampling strategy for IDIs and the IDI sample composition remain unclear. Who was invited and who took part (age, gender)?

**Answer:** The FGDs were stratified into groups of women, men, and teenagers to capture diverse perspectives within the community while minimizing external influences such as age and gender. This approach ensured that all significant groups had a voice. Regarding the IDIs, all invited participants had the opportunity to take part, and those who accepted were included, following the principle of theoretical saturation. Information on age and gender has been added as per the reviewer’s suggestion for improvement (lines 277–280).

**Research team:** Authors have introduced the research team. The team is rather big (compared to the study). Kindly clarify in the text the role of each team member in the study. This can be through initials throughout the Methods chapter.

**Answer:** The information was included (line 884-916).

**Results**

**Reviewer #1:** Please use data visualization tools to present the table content in a more intuitive and informative way.

**Answer:** We have incorporated data visualization tools to present the table content in a more intuitive and informative way, as suggested (lines 197–199).

**Reviewer #3:** Presentation/ structure: Whilst integration of results was recommended, authors decided to keep the quantitative and qualitative findings separate. Sub-themes improve structure.

Quotes: The quotes are well labelled. Changes may be high that participants can be identified because of the detailed information given and the small size of the community (64 people). Can identities be better protected?

**Answer:** Thank you for bringing this to us; to protect better the identity of participants, we removed the age.

**Conclusions**

**Reviewer #1:** The revised manuscript is a major improvement over the old version and has basically solved most of the problems I raised previously, and I recommend that it be adopted for publication.

**Reviewer #3:** Discussion: Authors have made changes. A introduction paragraph linking back to the research gap and question is still needed.

**Answer:** The information was included (line 129-133).

**Limitations:** More critical engagement with the drop in the number of participants from survey (n=42) to community meeting (n=20) is needed. The study was researcher-led whilst aiming at actively involving participants. Any recommendations for future research on how to mitigate against drop out?

**Answer:** The information was included (line 844-856).

**Conclusion:** As stated before, the messages in the Conclusion should be focused on the research aim and data – relative importance of SBE and improved access to healthcare in the riverine community, not the participatory methods. Kindly highlight the empirical results and new knowledge gained from the study. A critical evaluation of the method should be offered in a separate manuscript.

**Answer:** The conclusion was improved (line 859-873).

**Editorial and Data Presentation Modifications?**

Use this section for editorial suggestions as well as relatively minor modifications of existing data that would enhance clarity. If the only modifications needed are minor and/or editorial, you may wish to recommend “Minor Revision” or “Accept”.

**Reviewer #1:** Accept.

**Reviewer #3:** Minor Revision

**Summary and General Comments**

Use this section to provide overall comments, discuss strengths/weaknesses of the study, novelty, significance, general execution and scholarship. You may also include additional comments for the author, including concerns about dual publication, research ethics, or publication ethics. If requesting major revision, please articulate the new experiments that are needed.

**Reviewer #1:** The revised manuscript is a major improvement over the old version and has basically solved most of the problems I raised previously, and I recommend that it be adopted for publication. However, the following issues should be noted in the subsequent publication.

**1.** the use of data visualization tools to present the contents of the tables in a more intuitive and informative way.

**Answer:** The information was improved (197-199).

**2.** the consistency of language presentation in the manuscript, avoiding colloquial expressions and standardizing American or British spelling.

**Answer:** The manuscript was reviewed.

**3.** ensure that the research is cutting-edge and authoritative, ensure that more than 75% of the references come from Clarivate and Scopus databases, and do not cite withdrawn papers.

**Answer:** The references were reviewed.

**Reviewer #3:** The manuscript has been improved. Authors should focus on the empirical results. The evaluation of the methods should be reported in a separate manuscript and not mixed with the Results.

**Answer:** Thank you for the suggestion, we will make a separate manuscript.
